# Which Seems to Be Worst? Pain Severity and Quality of Life between Patients with Lateral Hip Pain and Low Back Pain

**DOI:** 10.1155/2018/9156247

**Published:** 2018-10-22

**Authors:** Raúl Ferrer-Peña, César Calvo-Lobo, Ramón Aiguadé, Josué Fernández-Carnero

**Affiliations:** ^1^Physical Therapy Department and Motion in Brains Research Group, Instituto de Neurociencias y Ciencias del Movimiento (INCIMOV), Centro Superior de Estudios Universitarios La Salle, Universidad Autónoma de Madrid, Madrid, Spain; ^2^Centro de Salud Entrevías, Gerencia de Atención Primaria, Servicio Madrileño de Salud, Madrid, Spain; ^3^Escuela Internacional de Doctorado, Department of Physical Therapy, Occupational Therapy, Rehabilitation and Physical Medicine, Universidad Rey Juan Carlos, Alcorcón, Spain; ^4^Nursing and Physical Therapy Department, Faculty of Health Sciences, Universidad de León, Ponferrada, León, Spain; ^5^Departamento de enfermería y fisioterapia, Universitat de Lleida, Lleida, Spain; ^6^Department of Physical Therapy, Occupational Therapy, Rehabilitation and Physical Medicine, Universidad Rey Juan Carlos, Alcorcón, Spain

## Abstract

**Purpose:**

The aim of this study was to compare the pain severity, health-related quality of life (HRQoL), and risk of continue having pain with prognostic risk scores (PRS), between patients referring greater trochanteric pain syndrome (GTPS) and chronic low back pain (CLBP).

**Methods:**

A descriptive, cross-sectional design using nonprobability convenience sampling was performed. A total sample of 102 patients were recruited from two primary health-care centers and divided into GTPS (*n* = 51) and CLBP (*n* = 51) groups. The primary outcome was pain severity which was assessed with the Spanish version of the Graded Chronic Pain Scale (GCPS). The secondary outcome was the HRQoL which was measured using the Spanish version of EuroQoL Five Dimensions and Five Levels (EQ-5D-5L) as well as the PRS.

**Results:**

Significant differences (P<0.05) were found within both groups in the pain severity global score with a medium effect size showing greater values for the CLBP group with regards to the GTPS group. The PRS in both groups did not show statistical differences (P>0.05). Nevertheless, subjects referring CLBP showed greater levels in the PRS than patients with GTPS. Comparing both groups, the HRQoL showed statistical differences (P<0.05) in the “pain/discomfort” domain in the CLBP group with respect to the GTPS group, but not in the other domains.

**Conclusions:**

Patients who suffered from CLBP showed greater pain severity and HRQoL discomfort with regard to patients with GTPS. Despite greater scores for CLBP, the PRS did not seem to be different between both conditions.

## 1. Introduction

Worldwide, chronic low back pain (CLBP) may be considered as one of the main musculoskeletal conditions, which causes disability for the life years [1]. CLBP is frequently referred to primary care and physical therapy units [2]. Up to 40% of the population will experience CLBP [3]. The CLBP prevalence increased during the last years with older age distributions and may be associated with the increase of psychological factors such as anxiety or depression [4–6]. Pain severity, disability, and health-related quality of life (HRQoL) did not seem to be associated with degenerative radiological modifications of the lumbar spine [7]. The socioeconomic factors may predict the CLBP disability process more than medical-biological characteristics and generate a strong impact in the Spanish primary care [8].

Recently, the greater trochanteric pain syndrome (GTPS) appeared as a common nonosteoarthritic hip condition reported usually in the primary health-care services [9–11]. This syndrome comprised signs and symptoms such as a lateral hip pain history as well as pain on lateral hip palpation, among other factors [9]. The GTPS prevalence reached 20.2% of the patients referred from primary care to a tertiary care orthopedic spine center. This syndrome was more commonly reported in women than in men, and the magnetic resonance imaging was not frequently associated with neurological findings [12]. In Spain, there is a lack of GTPS prevalence studies, and this may be secondary to the differential diagnosis challenge of lateral hip pain [10]. Among the musculoskeletal conditions, the GTPS may interfere with the HRQoL and the pain severity of patients referred from the primary health-care system [13].

Indeed, GTPS comprised patients' substantial proportion referred from primary health care in order to evaluate CLBP. Primary care physicians should be able to stablish an early diagnosis and prognosis in order to reduce costly patient referrals and avoid unnecessary surgery [12, 14]. GTPS and CLBP may reduce HRQoL and increase the pain severity as well as complicate the prognosis of primary care patients [13]. Indeed, CLBP was shown to be the musculoskeletal condition which generated the greater disability adjusted by life years impairing the healthy life expectancy as well as high chronic pain severity and HRQoL impairment [1, 13]. Nevertheless, up to date prior studies have not specifically compared both common conditions with regard to HRQoL, pain severity, and their prognostic risk scores (PRS) in primary care environments. Although chronic musculoskeletal conditions showed a clearly detrimental effect on the HRQL and the burden of musculoskeletal conditions has been compared with other common chronic diseases [13], the comparison of pain severity levels and quality of life between patients groups with CLBP and GTPS needs to be addressed. We hypothesize that patients with CLBP may show a higher pain severity, a greater impairment of HRQoL, and a higher risk of continue having pain with worse PRS than patients who suffer from GTPS. Therefore, the aim of the present study was to compare the pain severity, HRQoL, and risk of continue having pain with PRS measures, between patients referring GTPS and CLBP.

## 2. Methods

### 2.1. Study Design

A descriptive, cross-sectional design using nonprobability convenience sampling was performed. All participants were recruited since September 2016 to February 2017 and diagnosed as GTPS or CLBP by the referenced general practitioner from two primary health-care centers in Madrid, Spain, and were derived to the physical therapy unit. All the subjects gave their written informed consents. This study was accepted by the Southeast Local Research Committee of the Primary Health Care Management (Code 16/15). The reporting of the study followed the “STrengthening the Reporting of OBservational studies in Epidemiology” (STROBE) guidelines [15].

### 2.2. Subjects

The inclusion criteria for the present study were (1) patients referring low back pain [5, 6] or (2) patients with unilateral lateral hip pain [16], (3) tenderness on palpation at the greater trochanter [9], (4) being diagnosed and derived by a general practitioner, and (5) having pain almost until last 3 months before the assessment time.

The exclusion criteria were (1) presence of other musculoskeletal injury, neurological, or systemic condition that could affect balance/gait [16], (2) cognitive impairment or psychiatric disease, or (3) having surgical or traumatic history or corticoid local injection in previous six months.

### 2.3. Data Collection

A sociodemographic questionnaire containing gender, age, height, weight, civil state, level of education, and pain intensity was self-reported by the study participants.

#### 2.3.1. Primary Outcome

*Pain Severity*. The pain severity was assessed by means of the Spanish version of the Graded Chronic Pain Scale (GCPS). This scale is a self-reported instrument consisting of two subscales; the first scale evaluates the pain intensity, and the second scale assesses the perceived disability. The scale is formed by a total of 8 items, 7 of them are 11 points as Likert format, and the other item evaluates the perpetuation of pain, asking the number of pain days in the previous 6 months. The Spanish version of the GCPS has proven to be a valid and reliable instrument for assessing the severity of chronic pain. Concretely, high internal consistency (Cronbach's *α* = 0.87) and intraclass correlation coefficient (ICC = 0.81) have been described [17]. The total score of the scale ranges from 0 to 70 points, the chronic pain perpetuation with the first item, and also have a graduation in five levels with its punctuation [18].

#### 2.3.2. Secondary Outcomes

*Health-Related Quality of Life (HRQoL)*. It was measured by the Spanish version of EuroQoL Five Dimensions and Five Levels (EQ-5D-5L). This self-reported questionnaire has been widely used in the literature to report perceived health quality of life in many conditions and translated into 130 languages [19–21]. The instrument consists of two elements, the first one is a 5-item questionnaire, one for each domain assessed (mobility, self-care, usual activities, pain/discomfort, and anxiety/depression), and five levels on each domain (no problems, slight problems, moderate problems, severe problems, and extreme problems). Patients were asked to fill only one level on each domain (1 to 5). The digits on each domain can be combined on a five digit number ranging from 11111 to 55555. Also, the EQ-5D-5L results can be interpreted by the *Sum Score* that it is a severity index obtained with the summation of the levels in each dimension of the instrument, subtracting 5 points and multiplying the result by 5. It results on a 0-to-100 range new scale where more points mean more severity. Another way to interpret the results on the EQ-5D-5L is by means of the Index (*EQ-Index*); this approach compares the values in the five dimensions with 3125 different hypothetic health states adjusted by country population, being the “0” value assigned to death and “1” to the perfect health status. Values less than 0 are considered in the index, being those statuses interpreted as “worse than being dead.”

The second part of the questionnaire is a vertical 20 cm Visual Analogue Scale (*EQ-VAS*) in which subjects are asked to self-rate their health, from 0 “The worst health you can Imagine” to 100 “The best health you can imagine.” Finally, the Spanish EQ-5D-5L presented minimal floor and ceiling effects (<3%) and a Cronbach's *α* of 0.86 [22].

*Prognostic Risk Score (PRS)*. The PRS is an instrument to determine the probability of having pain in the next years. This tool is based on the perspective of a chronic pain continuum health status, nor a unique state of the subjects who referred pain during 3 or 6 months (as the conventional definition of chronic pain). It was calculated for each subject with the rules proposed by Von Korff and Miglioretti [23] and used in other studies [24, 25] with the scoring method described in Table 1. The cut-points were also established in low risk, intermediate risk, possible risk over 50%, and probable risk over 80% for the probability of having pain in the next 5 years in primary health-care samples [23]. In addition, a high level of reliability was demonstrated for the classification of people at high risk (over 80% probability) for suffering from clinically significant back pain at follow-up [24, 25].

### 2.4. Pain Intensity

The average pain intensity in the last seven days was measured by means of a Visual Analogue Scale (VAS). This scale consists of a horizontal 100 mm line, in which the patients must indicate their pain intensity. At the left side of the line appears the text “no pain” and at the right side appears “worst possible pain.” This instrument had demonstrated its validity and reliability measuring the pain intensity, showing ICCs among pain scales from 0.65 to 0.88 with a median *r* of 0.74 [26, 27].

### 2.5. Sample Size

Sample size was calculated with the G^∗^Power 3.1.9.2 for Mac OS X (G^∗^Power© from University of Düsseldorf, Germany) to determine a sufficient sample size considering a one-tailed *t*-test with two groups and a medium effect size to achieve clinically relevant differences (*d* = 0.50) in the primary variable (pain severity) to obtain a statistical power of 80% using an *α* error of 0.05. Based on the aforementioned assumptions, we estimated a sample size of at least 102 subjects [28].

### 2.6. Data Analysis

Data analyses were performed on SPSS for Mac OS X, Version 22.0 (SPSS Inc., Chicago IL) with a 95% confidence interval (CI) and considering statistically significant differences if *P* value < 0.05. Parametric tests were used because the sample size (greater than 30 subjects per group) was sufficient to be supported by the central limit theorem [29]. The continuous variables are presented as mean and standard deviation (SD), and the categorical variables are presented as absolute numbers and relative frequency (i.e., percentages). A Student's *t*-test for independent samples was used to compare the quantitative outcomes between subjects with GTPS and CLBP, and the chi-square test was used for the categorical ones. Furthermore, the effect sizes of the primary outcomes were calculated based on the following formula $d=2t/\sqrt{gdl}$, which is determined by the SD of the groups. Cohen's *d* size effect may be interpreted as slight (*d* lower than 0.20), fair (*d* from 0.20 to 0.49), moderate (*d* from 0.50 a 0.79), or large (*d* equal or higher than 0.80) [30].

## 3. Results

### 3.1. Sociodemographic Characteristics

A total sample of one hundred and two subjects were analyzed in this study and divided into two groups, one group of patients diagnosed of GTPS (*n* = 51) and another group of patients with CLBP (*n* = 51). Sociodemographic variables did not show any statistically significant difference (*P* > 0.05) between both groups. Descriptive variables of both groups are presented in Tables 1 and 2.

### 3.2. Primary Outcome

Regarding Student's *t*-test, a statistical significant difference (*P* < 0.05) was found between both groups in the pain severity global score with a medium effect size (mean difference = −7.49; CI 95% from −13.35 to −1.62; *d* = 0.51) showing greater values of the CLBP group with regard to the GTPS group. The results of the comparison for the other quantitative variables are presented in Table 3.

### 3.3. Secondary Outcomes

**Figure 1:**
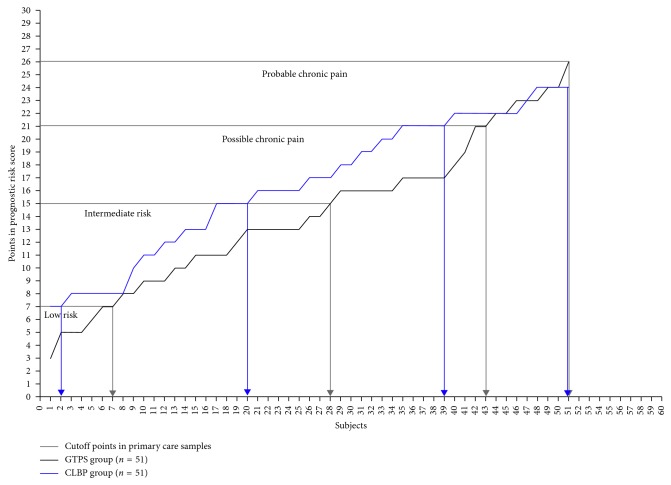
Number of subjects and the relation between GTPS and CLBP patients in PRS. CLBP, chronic low back pain; GTPS, greater trochanteric pain syndrome; PRS, prognostic risk scores.

The chi-square test comparing the PRS in both groups did not show statistical differences, as presented in Table 4. Nevertheless, subjects referring CLBP showed greater levels in the PRS than patients with GTPS. Levels of both groups with the cutoff points for primary health-care samples are presented in Table 4 and Figure 1.

Moreover, the chi-square test comparing the HRQoL between groups showed statistical differences (*P* < 0.05) in the “pain/discomfort” domain of the CLBP group with respect to the GTPS group, but not in the other domains, as presented in Table 5.

## 4. Discussion

To the authors' knowledge, this may be considered as the first study comparing both common conditions, CLBP and GTPS. CLBP seemed to show greater pain severity and HRQoL discomfort with respect to GTPS. The prognostic risk seemed to be similar between both musculoskeletal pathologies. Despite this, there is a tendency towards poorer quality of life, higher pain severity, and prognostic risk in the patients who suffer from CLBP with regard to patients who suffer from GTPS. These findings coincide with prior research about musculoskeletal conditions related to quality of life and pain severity [1, 7–9, 12, 13].

Here, we show a challenge in the differential diagnosis between both conditions which may commonly be considered as comorbidities in patients referred to primary health-care and physical therapy units [2, 12–14]. Therefore, the palpation skills and the clinical reasoning of physicians and physical therapists should be improved in order to diagnose and classify these primary care patients, avoid unnecessary costs, and provide interventions according to the HRQoL discomfort and pain severity [9–11, 13].

### 4.1. Implications for Clinical Practice

Although both CLBP and GTPS conditions may be focused as main musculoskeletal disorders for treating in primary health-care environments [12, 14], patients who suffer from CLBP need greater primary care attention to reduce pain severity and increase HRQoL with regard to patients who suffer from GTPS. Interventions such as multidisciplinary rehabilitation approaches should be prioritized in this kind of patients who attend to primary health-care units [31].

### 4.2. Limitations

Several limitations and methodological aspects should be taken into account regarding the present research. First, physical factors, such as pressure pain thresholds [32], recurrence or physical disability [7], were not assessed. Second, age distributions were not evaluated and may influence psychological aspects [5]. Third, acute and subacute low back pain or GTPS were excluded to include the central sensitization, which commonly occurs in a longer-term process [9, 17, 33]. Although the presence of prior surgeries was an inclusion criterion, former pain experiences of the subjects were not collected and should be considered for future research studies. Finally, individuals from various countries different from Spain and larger sample sizes may be beneficial to reach a study power improvement and find variations among countries [1].

## 5. Conclusions

Patients who suffered from CLBP showed greater pain severity and HRQoL discomfort with regard to patients with GTPS. Despite greater scores for CLBP, the PRS did not seem to be different between both conditions.

## Figures and Tables

**Table 1:** Description of quantitative sociodemographic variables between GTPS and CLBP groups.

	GTPS group (*n* = 51)	CLBP group (*n* = 51)	*P* value (Student's *t*-test)
	Mean ± SD	Mean ± SD	
Age (years)	48.88 ± 8.52	52.43 ± 12.34	0.095
BMI	26.89 ± 5.53	29.03 ± 5.60	0.054
Pain days, last 6 months	119.31 ± 62.21	136.03 ± 53.80	0.330
Pain intensity (VAS)	35.19 ± 23.56	40.10 ± 23.91	0.150
Chronicity (months)	28.52 ± 47.04	58.22 ± 90.44	0.065

GTPS, greater trochanteric pain syndrome; CBLP, chronic low back pain; SD, standard deviation; BMI, body mass index.

**Table 2:** Description of categorical sociodemographic variables between GTPS and CLBP groups.

	GTPS group (*n* = 51), *n* (%)	CLBP group (*n* = 51), *n* (%)	*P* value (chi-square test)
Gender			
Male	9 (17.6%)	18 (35.3%)	0.463
Female	42 (82.4%)	33 (64.7%)	

Civil state			
Single	12 (23.5%)	9 (17.6%)	0.218
Married	33 (64.7%)	28 (54.9%)	
Divorced	4 (7.8%)	7 (13.7%)	
Widower	2 (3.9%)	7 (13.7%)	

Education level			
None	0 (0%)	2 (3.9%)	0.347
Primary	15 (29.4%)	19 (37.3%)	
Secondary	18 (35.3%)	17 (33.3%)	
University	18 (35.3%)	13 (25.5%)	

All data are presented as number and percentage (n(%)). GTPS, greater trochanteric pain syndrome; CLBP, chronic low back pain; ^*∗*^*P* < 0.05.

**Table 3:** Comparison of quantitative result variables between GTPS and CLBP groups.

	GTPS group (*n* = 51)	CLBP group (*n* = 51)	Mean differences	95% of CI			Effect size (Cohen's *d*)
	Mean ± SD	Mean ± SD					
EuroQoL-5D value index	0.683 ± 0.200	0.689 ± 0.201	2.02	−6.34	To	10.38	0.03
EuroQoL-5D VAS	66.73 ± 22.96	64.70 ± 19.45	−0.006	−0.085	To	0.072	0.10
EuroQol-5D sum score	23.03 ± 14.56	24.31 ± 14.52	−1.27	−6.98	To	4.43	0.08
GCPS total score	28.11 ± 14.74	35.60 ± 15.11	−7.49	−13.35	To	−1.62	0.51^*∗*^
Prognostic risk score	14.21 ± 5.78	16.47 ± 5.34	−2.25	−4.44	To	−0.06	0.42

SD, standard deviation; CI, confident interval; VAS, Visual Analogue Scale; GCPS, Graded Chronic Pain Scale; GTPS, greater trochanteric pain syndrome; CBLP, chronic low back pain; ^*∗*^*P* < 0.05.

**Table 4:** Description of the prognostic risk score Categories between GTPS and CLBP groups.

Prognostic risk score	GTPS group (*n* = 51)	CLBP group (*n* = 51)	*P* value (chi-square test)
Low risk	7 (13.7%)	2 (3.9%)	0.233
Intermediate risk	21 (41.2%)	18 (35.3%)	
Possible chronic pain (50% risk)	15 (29.4%)	19 (37.3%)	
Probable chronic pain (80% risk)	8 (15.7%)	12 (23.5%)	

GCPS, Graded Chronic Pain Scale; GTPS, greater trochanteric pain syndrome; CBLP, chronic low back pain.

**Table 5:** Descriptive data and of five dimensions of EQ-5D and comparison between the GTPS and CLBP groups.

EQ-5D dimension		Groups		*P* value (chi-square test)
		GTPS group (*n* = 51)	CLBP group (*n* = 51)	
Mobility	No problem	16 (31.4%)	17 (33.3%)	0.308
	Slight problem	19 (37.3%)	23 (45.1%)	
	Moderate problem	13 (25.5%)	10 (19.6%)	
	Severe problem	3 (5.9%)	0 (0%)	
	Unable to	0 (0%)	1 (2%)	

Self-care	No problem	38 (74.5%)	38 (74.5%)	0.783
	Slight problem	6 (11.8%)	6 (11.8%)	
	Moderate problem	7 (13.7%)	6 (11.8%)	
	Severe problem	0 (0%)	1 (2%)	
	Unable to	0 (0%)	0 (0%)	

Usual activities	No problem	18 (25.3%)	23 (45.1%)	0.436
	Slight problem	23 (45.1%)	15 (29.4%)	
	Moderate problem	9 (17.6%)	12 (23.5%)	
	Severe problem	1 (2%)	1 (2%)	
	Unable to	0 (0%)	0 (0%)	

Pain/discomfort	No problem	4 (7.8%)	1 (2%)	0.024^*∗*^
	Slight problem	21 (41.2%)	13 (25.5%)	
	Moderate problem	16 (31.4%)	31 (60.8%)	
	Severe problem	10 (19.6%)	6 (11.8%)	
	Unable to	0 (0%)	0 (0%)	

Anxiety/depression	No problem	31 (60.8%)	21 (41.2%)	0.052
	Slight problem	13 (25.5%)	18 (35.3%)	
	Moderate problem	2 (3.9%)	10 (19.6%)	
	Severe problem	3 (5.9%)	1 (2%)	
	Unable to	2 (3.9%)	1 (2%)	

All data are presented as number and percentage (*n* (%)). EQ-5D, EuroQoL Five Dimensions; GTPS, greater trochanteric pain syndrome; CBLP, chronic low back pain.

## Data Availability

The data used to support the findings of this study are available from the corresponding author upon request.

## Abbreviations

BMI: Body Mass Index
GCPS: Graded Chronic Pain Scale
EQ-5D: EuroQoL Five Dimensions
GTPS: Greater Trochanteric Pain Syndrome
CBLP: Chronic Low Back Pain
SD: Standard Deviation
CI: Confident Interval
VAS: Visual Analogue Scale.
